# Individuals with Peripheral Artery Disease (PAD) and Type 1 Diabetes Are More Likely to Undergo Limb Amputation than Those with PAD and Type 2 Diabetes

**DOI:** 10.3390/jcm9092809

**Published:** 2020-08-31

**Authors:** Nidhi Jain, Manyoo A. Agarwal, Diana Jalal, Ayotunde O. Dokun

**Affiliations:** 1 Torrance Memorial Physician Network, Torrance, CA 90505, USA; nidhij50@gmail.com; 2 Department of Internal Medicine, University of Tennessee Health Science Center, Memphis, TN 38163, USA; 3 Division of Cardiovascular Medicine, University of California Los Angeles, Los Angeles, CA 90095, USA; 4 Division of Nephrology, Department of Medicine, Carver College of Medicine, University of Iowa, Iowa, IA 52242, USA; diana-jalal@uiowa.edu; 5 Division of Endocrinology and Metabolism, Department of Medicine, Carver College of Medicine, University of Iowa, Iowa, IA 52242, USA

**Keywords:** amputation, type 1 diabetes, peripheral artery disease, NIS, HCUP, type 2 diabetes, procedure, outcomes

## Abstract

Background: Limited data exist comparing how type 1 diabetes mellitus (DM) and type 2 DM may have differential effects on peripheral artery disease (PAD) severity. We aimed to study the association of type of DM with the procedure utilized in hospitalizations with a diagnosis of PAD. Methods: We used the national inpatient sample databases from 2003 to 2014 to identify hospitalizations with a diagnosis of PAD and type 1 or type 2 DM. Logistic regression was utilized to evaluate the association between type of DM and procedure utilized (amputation-overall, major, endovascular revascularization, surgical revascularization). Results: We identified 14,012,860 hospitalizations with PAD diagnosis and DM, 5.6% (*n* = 784,720) had type 1 DM. The patients with type 1 DM were more likely to present with chronic limb-threatening ischemia (CLTI) (45.2% vs. 32.0%), ulcer (25.9% vs. 17.7%), or complicated ulcer (16.6% vs. 10.5%) (all *p* < 0.001) when compared to those with type 2 DM. Type 1 DM was independently and significantly associated with more amputation procedures (adjusted odds ratio = 1.12, 95% confidence interval [CI] I 1.08 to 1.16, *p* < 0.001). Overall, in-hospital mortality did not differ between the individuals with type 1 and type 2 DM. The overall mean (95% CI) length of stay (in days) was 6.6 (6.5 to 6.6) and was significantly higher for type 1 DM (7.8 [7.7 to 8.0]) when compared to those with type 2 DM (6.5 [6.4 to 6.6]). Conclusion: We observed that individuals with PAD and type 1 DM were more likely to present with CLTI and ulcer and undergo amputation when compared to those with PAD and type 2 diabetes. Further studies are needed to better understand the underlying mechanisms behind these findings and to identify novel interventions to reduce the risk of amputation in patients with type 1 DM.

## 1. Background

Peripheral artery disease (PAD) is now recognized to have a prevalence that is similar to that of ischemic heart disease [1,2,3,4] and affects about 3% to 10% of adults in the world [4,5,6]. PAD is caused by atherosclerosis in the vast majority of patients and the most common site is the lower extremity where occlusive disease leads to impaired perfusion [1,3,7]. PAD can have different severity of clinical presentations such as intermittent claudication, presence of ulcer, gangrene, complicated infections (osteomyelitis, cellulitis), and chronic limb-threatening ischemia (CLTI) [1,7,8,9,10,11].

Individuals with diabetes mellitus (DM) are five times more likely to develop CLTI than those with PAD without DM [7]. Both type 1 and type 2 DM are associated with increased risk of developing PAD and increased PAD severity [12,13,14,15]. Although both forms of diabetes have hyperglycemia as a key metabolic abnormality [13,14,15], in type 1 DM insulin deficiency is the primary cause of hyperglycemia while in type 2 DM hyperglycemia results from insulin resistance, impaired insulin signaling, and, in some individuals, impaired insulin secretion is also a contributing factor [15,16]. These differences have caused investigators to hypothesize that the molecular mechanisms driving the development and severity of PAD may not be due solely to hyperglycemia and may differ in type 1 and type 2 DM [8,17]. Several studies have investigated gene expression in the ischemic tissues following experimental PAD in mice with type 1 or type 2 diabetes and they report major differences in the molecular pathways predicted to be impacted by diabetes [8,17]. These findings suggest the molecular mechanisms by which type 1 DM contributes to poor PAD outcomes may differ from the mechanism by which type 2 DM contributes to poor PAD outcomes. Given these findings, we hypothesized that type 1 and type 2 diabetes may have differential effects on PAD severity. We tested this hypothesis by analyzing data from the National (Nationwide) Inpatient Sample (NIS) data and compared characteristics, treatment, and outcomes for individuals with PAD and DM who were admitted from 2003 to 2014.

## 2. Methods

### 2.1. Data Source

Data for this analysis were obtained from NIS. The NIS, part of HealthCare Utilization Project (HCUP) sponsored by the Agency for Healthcare Research and Quality (AHRQ), contains stratified samples of ~20% of U.S. hospital discharge data and has been used previously to study outcomes, temporal trends, and national estimates [18,19,20]. It is the largest all-payer, inpatient-care database in the United States and details are available elsewhere. [21,22,23] The database contains de-identified information regarding each hospitalization, including demographic characteristics, comorbidities, discharge diagnoses, procedure codes, and discharge disposition. Patients admitted under observation status and those admitted to short-term rehabilitation hospitals, long-term non-acute care hospitals, psychiatric hospitals, and alcoholism or chemical dependency units are not included. The internal and external validity of the NIS database are maintained through annual data quality assessments and comparison with other databases, such as the National Hospital Discharge Survey and MedPAR (Medicare Provider and Analysis Review). These reports are published on the NIS website (http://www.hcup-us.ahrq.gov/db/nation/nis/nisrelatedreports.jsp).

### 2.2. Study Population

**Figure 1:**
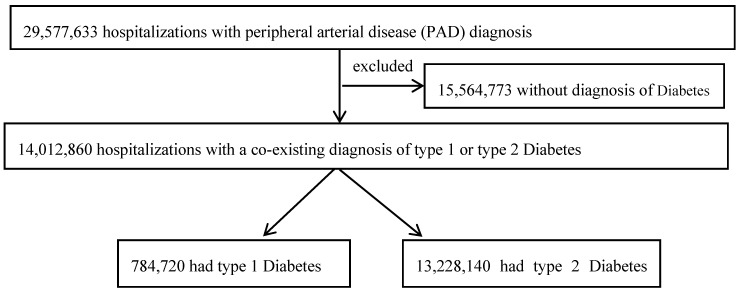
Flowchart describing identification of the study cohort.

Using data from 2003 to 2014, we identified hospitalizations with any primary diagnosis code (reason for admission) or secondary diagnosis code indicating PAD of the extremities (*n* = 29,577,633). The diagnoses had been coded based on the International Classification of Diseases-Ninth Edition-Clinical Modification (ICD-9-CM) codes, as done previously [19,20]. We then identified those with a diagnosis of either type 1 (*n* = 784,720) or type 2 DM (*n* = 13,228,140) (Figure 1) [24]. The ICD-9 codes are detailed in Supplemental Tables S1–S3.

### 2.3. Definition of PAD-Related Procedures

This was done using ICD-9 procedure codes (Supplemental Table S3) for the following procedures: Open revascularization (endarterectomy, aortoiliacfemoral bypass, or infrainguinal bypass), endovascular revascularization (angioplasty, stenting, atherectomy, or mechanical thrombectomy), and amputations (minor and major, below or above knee).

### 2.4. Definition of PAD-Related Disease Severity

This was done using ICD-9 diagnosis codes (Supplemental Tables S1 and S2) for the following characteristics: Intermittent claudication (IC), critical limb-threatening ischemia (CLTI), ulcer, and complicated ulcer (defined as the presence of ulcer along with one of the following: Gangrene, osteomyelitis, and cellulitis).

### 2.5. Statistical Analysis

We followed the recommendations from the AHRQ for analysis using survey data such as using survey-specific statements and utilizing patient-specific and hospital-specific discharge weights [21]. Estimates were weighted, unless otherwise noted, to allow for nationally representative interpretations and to account for the 2012 changes to the NIS sampling strategy [21]. We accounted for hospital-level clustering of patients in the sampling design. We described characteristics of the sample, including age, sex, race/ethnicity, primary payer status, type of presentation (CLTI or IC), and presence of ulcer, overall and complicated) (Table 1) and clinical comorbidities (hypertension, dyslipidemia, smoking, history of myocardial infarction, history of coronary artery bypass graft, history of percutaneous coronary intervention, heart failure, atrial fibrillation, chronic pulmonary disease, prior cerebrovascular disease (CVA), chronic kidney disease (CKD), end-stage renal disease (ESRD), and carotid artery disease (CAD)) (Table 1). The comorbidities not provided as part of the dataset were identified using ICD-9 codes (Supplemental Table S1). Age was defined as age groups 18–40, 41–60, 61–75, and 75 and above. The details about AHRQ comorbidities provided in NIS dataset along with a combination of ICD-9-CM codes and HCUP Clinical Classification Software codes were used to identify clinical comorbidities as done previously [25,26,27]. Data points were missing for the following variables: Race (14%), primary payer status (0.2%), and chronic comorbidities (0.5%). Of note, all analyses were conducted twice, once without exclusion of subjects and then again excluding those with missing variables. The results were similar for both analyses, and the data presented in the manuscript included the full cohort (did not exclude the subjects with missing variables).

**Figure 2:**
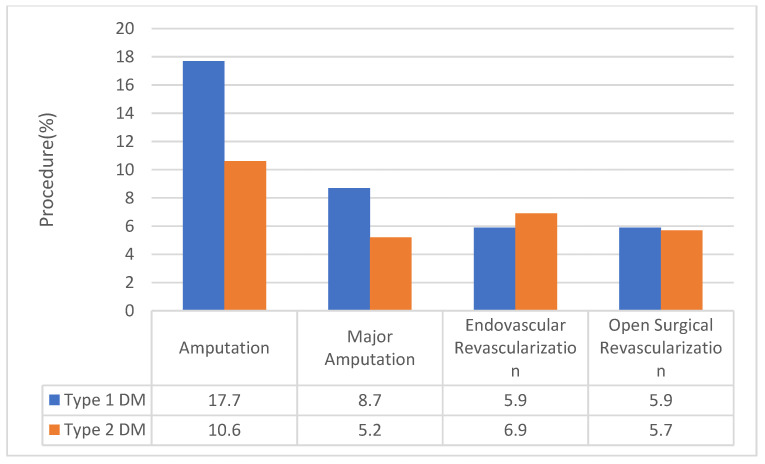
Revascularization procedures utilized based on type of diabetes (type 1 vs. type 2 DM). *Y*-axis indicates the % of individuals with type 1 or type 2 DM and PAD undergoing a given procedure. The *X*-axis indicates the type of procedure performed. DM, diabetes mellitus.

**Figure 3:**
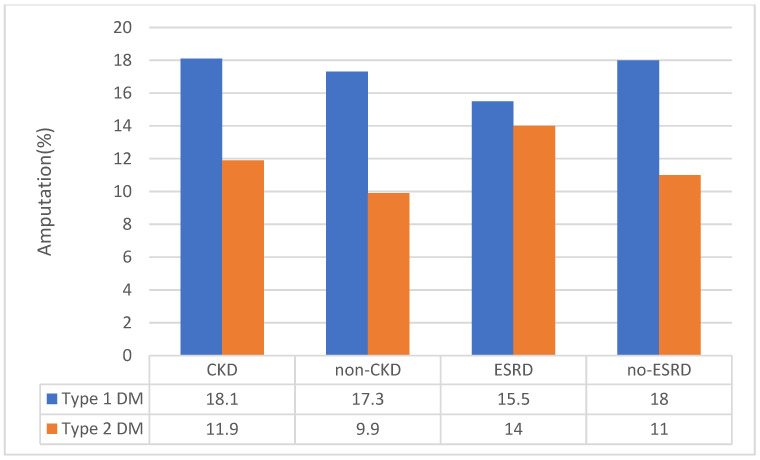
Proportion of type 1 and type 2 diabetes patients based upon presence and absence of CKD and ESRD that underwent amputation procedures. CKD, chronic kidney disease; DM, diabetes mellitus; ESRD, end-stage renal disease.

**Figure 4:**
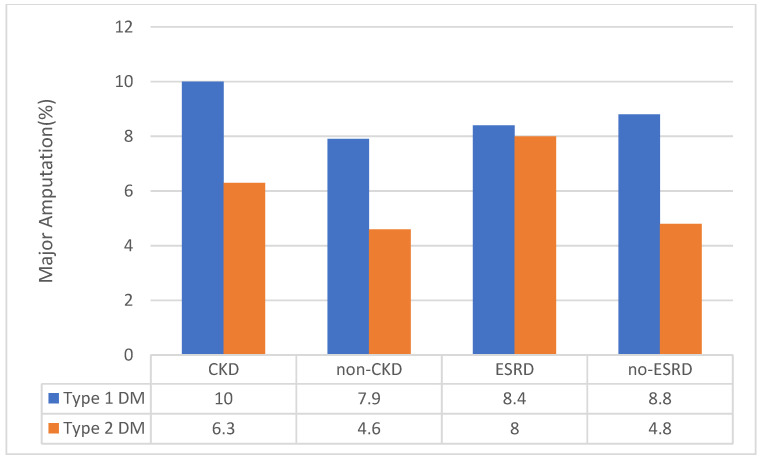
Proportion of type 1 and type 2 diabetes patients based upon presence and absence of CKD and ESRD that underwent major amputation procedures. CKD, chronic kidney disease; DM, diabetes mellitus; ESRD, end-stage renal disease.

Characteristics of the study population are summarized for type 1 and type 2 DM groups, and presented as frequency (percent, 95% confidence interval (CI)) for categorical variables and mean ± standard deviation (SD)/95% CI for continuous variables. Pearson χ^2^ test and student’s *t*-test were used to describe the characteristics of hospitalizations between type 1 DM and type 2 DM. Cross-sectional analysis was performed using logistic regression with DM type as a categorical variable. Adjusted odds ratios (AORs) and 95% CI were used to report the results of logistic regression. We used multivariable logistic regression models to compare type of procedure utilized between type 1 and type 2 DM (Figure 2). We studied the association of type of diabetes with the type of procedure utilized during hospitalization in four different regression models using covariates selected based upon theoretical considerations and priori (Table 2): Model 1, unadjusted; Model 2, age, sex, race/ethnicity, primary payer status; Model 3, model 2 plus calendar year, smoking, hypertension, hyperlipidemia, history of myocardial infarction, carotid artery disease, history of coronary revascularization, atrial fibrillation, heart failure, chronic pulmonary disease, CKD, and ESRD; Model 4, Model 3 plus presence of CLTI, IC, and complicated ulcer. (Supplemental Tables S4 and S5) Additionally, we observed a significant interaction with CKD diagnoses and conducted sensitivity analysis with and without CKD (Figure 3 and Figure 4). A subgroup analysis was performed to analyze the outcome of amputation for those hospitalizations with and without ESRD based upon type of DM. Lastly, a subgroup analysis after excluding all patients with intermittent claudication was also performed (Figure 3 and Figure 4). Study was deemed exempt by the University of Tennessee Health Science Center Institutional Review Board. All analyses were conducted with Stata/MP version 15.1 (StataCorp LLC, College Station, TX, USA) and SPSS version 23.0 (IBM Corp., Armonk, NY, USA). We utilized svy-suite extension in STATA. A two-sided *p* < 0.001 was considered to be statistically significant.

## 3. Results

### 3.1. Clinical Characteristics According to DM Status

Among an overall 14,012,860 admissions with a diagnosis of PAD and DM, 5.6% (*n* = 784,720) had Type 1 DM. Overall, Type 1 DM patients were younger, more likely to be white males, have private insurance, and less likely to have concurrent diagnoses of hypertension, smoking, dyslipidemia, history of myocardial infarction, history of coronary revascularization procedures, congestive heart failure, atrial fibrillation, cerebrovascular disease, and chronic pulmonary disease (Table 1). Individuals with type 1 DM had significantly higher concurrent diagnoses of CKD (Table 1). Importantly, the individuals with type 1 DM were more likely to present with CLTI (45.2% vs. 32.0%), ulcer (25.9% vs. 17.7%), and complicated ulcer (16.6% vs. 10.5%) (all *p* < 0.001) when compared to type 2 DM (Table 1).

### 3.2. Vascular Procedure According to DM Status

Patients with type 1 DM were more likely to undergo all types of amputations (17.7% vs. 10.6%) and major amputations (8.7% vs. 5.2%) compared to patients with type 2 DM (Table 1 and Figure 2). This association was independent of demographics, other risk factors for PAD, and presentation severity as shown in Table 2 (the AOR for type 1 DM undergoing amputation vs. type 2 DM = 1.12, 95% CI 1.08 to 1.16; the AOR for type 1 DM undergoing major amputation vs. type 2 DM = 1.15, 95% CI = 1.11 to 1.20). Patients with type 1 DM were, additionally, less likely to undergo endovascular revascularizations (5.9% vs. 6.9%) (Figure 2). Logistic regression analysis revealed the association was also independent from confounding variables (AOR = 0.84, 95% CI 0.79 to 0.88) (Table 2). Similar results were obtained after excluding those with a diagnosis of intermittent claudication.

### 3.3. Sensitivity Analysis for CKD Status

This was considered since we observed that patients admitted with PAD and type 1 DM had significantly higher prevalence of CKD than those with PAD and type 2 DM, and it is known that CKD is an independent risk factor for PAD [28]. Among those with a diagnosis code of CKD, individuals with PAD and type 1 DM had higher amputation rates (18.1% vs. 11.9%) (Figure 3). Additionally, in individuals with type 1 DM and PAD without a diagnosis of CKD, type 1 DM was still associated with a higher amputation rate (17.3% vs. 9.9%) (Figure 4). Therefore, the presence or absence of CKD diagnosis did not influence the conclusion from our findings. On subgroup analysis, ESRD was associated with higher amputation procedures (14.1% vs. 10.6%) and major amputation rates (8.1% vs. 5.0%) for the overall cohort (Figure 3 and Figure 4). The association of ESRD with amputations persisted even after adjustment in regression analysis (Supplemental Tables S4 and S5).

### 3.4. Other Outcomes According to DM Status

Overall, in-hospital mortality was similar between the individuals with type 1 and type 2 DM (3.1%; 3.0% in type 1 vs. 3.1% in type 2 DM, *p* = 0.23). The overall mean (95% CI) length of stay (in days) was 6.6 (6.5 to 6.6) and was significantly higher for type 1 DM (7.8 [7.7 to 8.0]) when compared to those with type 2 DM (6.5 [6.4 to 6.6]). Overall median hospitalization charges ($) were $45,231 ($44,203 to $46,260); $45,050 ($43,474 to $46,626) for type 1 DM and $45,242 ($44,221 to $46,263) for type 2 DM.

## 4. Discussion

The goal of our study was to test the hypothesis that among individuals with PAD and diabetes, the type of diabetes the individual has, whether type 1 or type 2, would have a different impact on PAD severity. We analyzed data from the National Inpatient Sample, from 2003 to 2014, to identify individuals with PAD and type 1 or type 2 DM, and compared the amputation rates among those with PAD and type 1 DM to that in those with PAD and type 2 DM. Our results show individuals with a diagnosis of PAD and type 1 DM were more likely to present with CLTI, ulcer, and complicated ulcer. Also, type 1 DM patients with PAD were more likely to undergo amputation (17.7% vs. 10.6%) when compared to those with a diagnosis of type 2 DM.

This finding was quite unexpected, given that the individuals with PAD and type 2 DM were about 10 years older than those with PAD and type 1 DM. They were also more likely to have CAD (4.5% vs. 2.1%, *p* < 0.01), CVA (10.8% vs. 6.1%, *p* < 0.01), and cardiovascular risk factors such as smoking (24.4% vs. 16.6%, *p* < 0.01), hypertension (75.2% vs. 66.3%, *p* < 0.01), and dyslipidemia (45.4% vs. 30.2%, *p* < 0.01). There is a number of possible explanations for these findings. Individuals with type 1 DM tend to develop the disease at a younger age, on average, compared to those with type 2 DM and, therefore, those with PAD and type 1 DM may have been exposed to hyperglycemia for a longer duration when compared to those with type 2 DM. We speculate that a longer duration of hyperglycemia exposure in individuals with PAD may lead to increased PAD severity. Alternatively, the metabolic differences between type 1 and type 2 DM, rather than the duration of glycemic exposure, may explain the difference in PAD severity. Although hyperglycemia is common to both type 1 and type 2 DM, insulin deficiency is a major feature in type 1 DM while hyperinsulinemia and impaired insulin signaling is more typical in type 2 DM [29,30]. In our review of the literature, we did not identify a specific metabolic difference between type 1 and type 2 DM that could specifically explain our findings. However, we speculate that the chronic relative insulin deficiency in type 1 DM may contribute to impaired perfusion of the limb and increased susceptibility of the tissue to ischemic injury. Insulin may also be necessary for skeletal muscle adaptation to injury since studies in mice have shown increased skeletal muscle susceptibility to ischemic reperfusion injury in mice with type 1 DM [31]. Studies to specifically test the impact of insulin deficiency versus hyperinsulinemia on tissue adaptation to ischemia may provide insight into the metabolic differences in type 1 and type 2 DM driving this major observation. Interestingly, in recent preclinical studies where we investigated gene expression in mouse hind limbs following experimental PAD, although both type 1 and type 2 DM altered gene expression compared to nondiabetic mice, the top pathway affected by type 1 DM was different from the top pathways affected by type 2 DM [8,17]. For example, the cell cycle and DNA replication were the top pathways identified in type 1 DM while phagosome and lysosome pathway where the top pathways identified in the type 2 DM mice.

Another interesting finding in this study was that individuals with PAD and type 2 DM were more likely to have other cardiovascular risk factors, except for CKD, which was more prevalent in the individuals with PAD and type 1 DM (38.3% vs. 33.5%). This raised the possibility that the increased PAD severity in type 1 DM patients may be driven by higher likelihood of CKD in this group. However, when the data were analyzed, excluding individuals with CKD, individuals with PAD and type 1 DM were still more likely to require amputation than those with PAD and type 2 DM. In fact, even among those with CKD, individuals with PAD and type 1 DM were more likely to undergo amputation than individuals with PAD and type 2 DM. These data suggest that the higher risk of amputation in this study is due to type of DM rather than CKD. This conclusion is further supported by recent data from The Examining Use of Ticagrelor in PAD (EUCLID) trial, where CKD did not predict the risk of amputation in patients with PAD [32].

Our present study had several limitations that deserve mention, majorly due to the retrospective nature of study and utilization of an administrative database. We defined our data using ICD-9 codes based upon previously published PAD literature [19,20] and, thus, information regarding specific characteristics such as the location, laterality, character, severity, and extent of lesions were not available. These include lack of clinical details such as hemoglobin A_1c_ values, pharmacotherapeutic profile (use of insulin, anti-diabetic medications), and granular details about the ulcer (size, depth, etc.), neuropathy, and procedures. The unit of analysis was hospitalization and, given the lack of patient identifier details, information regarding readmissions and repeated procedures could not be delineated. Hence, patients with multiple admissions would be counted multiple times. The observational nature of the study did not enable casual inferences. The outcomes were limited to in-hospital events, did not contain details about long-term follow-up, and it was not possible to describe details about post-discharge events such as revascularization outcomes (e.g., patency, wound healing, etc.). Additionally, given the nature of the dataset, we were not able to stratify patients using classification systems such as Wound, Ischemia and foot Infection score [33]. The dataset was limited until calendar year 2014 and, hence, did not allow us to determine if our findings accurately reflected this patient population today. Nevertheless, NIS data provided an unequaled statistical power to examine the differences in treatment and outcomes of PAD-related procedural hospitalizations, and our study, to our knowledge, is the first to explore these possible differences between types of diabetes. In conclusion, the presence of type 1 DM in individuals with PAD is associated with increased likelihood of limb amputation. These findings may be due to longer duration of glycemic exposure or metabolic effects of insulin deficiency. Additional research is needed to further dissect the effects of the metabolic condition in type 1 and type 2 DM on PAD outcomes. In addition, novel therapies and interventions are needed to specifically target chronic limb-threatening ischemia and amputation in individuals with type 1 DM.

## Figures and Tables

**Table 1:** Demographics and comorbidities in PAD-related procedural hospitalizations for patients with type 1 and type 2 diabetes mellitus.

Variable	Overall (*n* = 14,012,860)	Type 1 DM (*n* = 784,720)	Type 2 DM (*n* = 13,228,140)	*p*-Value
**Age, mean ± SD (years)**	69.0 + 12.3	60.0 + 14.2	69.6 + 12.0	<0.001
**Age group (years)**				<0.001
18–40	1.5 (1.5 to 1.6)	9.8 (9.4 to 10.2)	10.0 (9.9 to 10.1)	
41–60	22.9(22.6 to 23.1)	41.2 (40.5 to 42.0)	21.8 (21.5 to 22.1)	
61–75	42.0 (41.9 to 42.2)	32.2(31.6 to 32.9)	42.6 (42.4 to 42.8)	
>75	33.6 (33.3 to 33.9)	16.8 (16.2 to 17.3)	34.6 (34.3 to 34.9)	
Females	43.3(43.1 to 43.5)	43.2(42.7 to 43.7)	43.3(43.1 to 43.5)	0.71
**Race/Ethnicity**				<0.001
Caucasian	65.7 (64.5 to 66.8)	65.6 (63.5 to 67.6)	65.7 (64.5 to 66.8)	
African American	17.7 (16.9 to 18.6)	19.8 (18.3 to 21.3)	17.6 (16.8 to 18.5)	
Others *	16.6(15.6 to 17.6)	14.6 (13.3 to 16.1)	16.7 (15.7 to 17.8)	
**Primary expected payer**				<0.001
Public insurance	81.9 (81.6 to 82.2)	76.0 (75.4 to 76.6)	82.2 (81.9 to 82.5)	
Private insurance	14.5(14.2 to 14.7)	19.9 (19.3 to 20.4)	14.1 (13.9 to 14.4)	
Others ^@^	3.6 (3.5 to 3.9)	4.1(3.9 to 4.4)	3.7 (3.5 to 3.9)	
**Comorbidities**				
Smoker	23.9 (23.5 to 24.4)	16.6 (16.0 to17.2)	24.4 (23.9 to 24.8)	<0.001
Hypertension	74.7 (74.4 to 75.0)	66.3(65.6 to 66.9)	75.2 (74.9 to 75.4)	<0.001
Dyslipidemia	44.6 (44.0 to 45.2)	30.2 (29.4 to 30.1)	45.4 (44.9 to 46.0)	<0.001
Prior myocardial infarction	11.5 (11.3 to 11.8)	9.4 (9.1 to9.8)	11.6 (11.4 to 11.9)	<0.001
Prior percutaneous coronary intervention	9.1 (8.9 to 9.3)	6.0 (5.7 to 6.3)	9.3(9.1 to 9.5)	<0.001
Prior coronary artery bypass grafting	15.7 (15.5 to 16.0)	12.8(12.4 to 13.2)	15.9 (15.7 to 16.1)	<0.001
Carotid Artery Disease	4.4 (4.3 to 4.5)	2.1(1.9 to 2.2)	4.5 (4.4 to 4.7)	<0.001
Prior cerebrovascular vascular accident	10.5 (10.3 to 10.7)	6.1 (5.8 to 6.3)	10.8 (10.5 to 11.0)	<0.001
Atrial fibrillation	17.3 (17.1 to 17.5)	9.2 (8.9 to 9.5)	17.8(17.6 to 18.0)	<0.001
Congestive Heart failure	19.4 (19.1 to19.6)	17.7(17.2 to 18.1)	19.5 (19.2 to 9.7)	<0.001
Chronic Pulmonary Disease	24.5 (24.2 to 24.8)	16.3(15.9 to 16.8)	25.0 (24.7 to 25.3)	<0.001
Chronic kidney disease	33.8(33.4 to 34.2)	38.3(37.6 to 39.1)	33.5 (33.1 to 33.9)	<0.001
End-Stage Renal Disease	11.6 (11.5 to 11.6)	13.4 (13.1 to 13.6)	11.5(11.4 to 11.5)	<0.001
**Disease severity**				
Intermittent Claudication	3.6(3.5 to 3.6)	2.2 (2.1 to 2.3)	3.6(3.6 to 3.7)	<0.001
CLTI	32.7 (32.4 to 33.1)	45.2(44.5 to 45.8)	32.0 (31.6 to 32.4)	<0.001
Ulcer	18.1 (17.9 to 18.3)	25.9(25.4 to 26.4)	17.7(17.4 to 17.9)	<0.001
Complicated Ulcer ^#^	10.9 (10.7 to 11.0)	16.6 (16.2 to 17.0)	10.5 (10.4 to 10.7)	<0.001
**Outcomes**				
Length of stay	6.6 (6.5 to 6.6)	7.8 (7.7 to 8.0)	6.5 (6.4 to 6.6)	<0.001
In-hospital mortality	3.1 (3.0 to 3.1)	3.0 (2.9 to 3.1)	3.1 (3.0 to 3.1)	0.23
Major or Minor Amputation	11.0 (10.8 to 11.2)	17.7 (17.2 to 18.1)	10.6 (10.4 to 10.8)	<0.001
Major Amputation	5.4 (5.3 to 5.5)	8.7 (8.5 to 9.0)	5.2(5.1 to 5.3)	<0.001
Endovascular revascularization	6.8 (6.6 to 7.1)	5.9 (5.6 to 6.2)	6.9(6.6 to 7.1)	<0.001
Open surgical revascularization	5.7 (5.5 to 5.8)	5.6 (5.6 to 6.2)	5.7 (5.5 to 5.8)	0.12

* Latino, Asian, Pacific Islander, unknown; ^@^ self-pay, uninsured, unknown; ^#^ ulcer plus one of the one following: Gangrene, osteomyelitis, cellulitis; PAD, peripheral artery disease, SD, standard deviation, CI, confidence interval; CLTI: chronic limb-threatening ischemia.

**Table 2:** Association of type 1 diabetes with the type of procedure utilized during hospitalization.

	*AOR for Vascular Procedure in Type 1 DM* *			
Vascular Procedure	Model 1	Model 2	Model 3	Model 4
Amputation (major or minor)	1.81 (1.77 to 1.87)	1.43 (1.39 to 1.48)	1.19 (1.16 to 1.23)	1.12 (1.08 to 1.16)
Major Amputation	1.77 (1.70 to 1.81)	1.54 (1.48 to 1.59)	1.22 (1.18 to 1.27)	1.15 (1.11 to 1.20)
Endovascular revascularization	0.85 (0.81 to 0.89)	0.79 (0.75 to 0.85)	0.81 (0.76 to 0.85)	0.84 (0.79 to 0.88)
Open revascularization	1.04 (0.99 to 1.09)	0.94 (0.89 to 0.99)	0.92 (0.87 to 0.96)	0.92 (0.88 to 0.96)

AOR, adjusted odds ratio; DM, diabetes mellitus; CI, confidence interval. * Type 2 DM was used as the reference. Data were adjusted for the following covariates: Model 1, unadjusted; Model 2, age, sex, race/ethnicity, primary payer status; Model 3, adjusted for age, gender, race, primary payer status, calendar year, smoking, hypertension, hyperlipidemia, history of myocardial infarction, carotid artery disease, history of coronary revascularization, atrial fibrillation, heart failure, chronic pulmonary disease, chronic kidney disease, and end-stage renal disease; Model 4, Model 3 plus presence of intermittent claudication, chronic limb-threatening ischemia, or complicated ulcer.

## Supplementary Materials

The following are available online at https://www.mdpi.com/2077-0383/9/9/2809/s1, Table S1: ICD-9 diagnosis codes for PAD diagnosis and other comorbidities, Table S2: ICD-9 diagnosis codes for Chronic Limb threatening Ischemia, Table S3: ICD-9 procedural codes, Table S4: The adjusted odds ratio and 95% confidence intervals of the covariates included in the multivariable unconditional logistic regression model for the outcome of Amputations, Table S5: The adjusted odds ratio and 95% confidence intervals of the covariates included in the multivariable unconditional logistic regression model for the outcome of Major Amputations.
