# Supplementary material for: Individuals with Peripheral Artery Disease (PAD) and Type 1 Diabetes Are More Likely to Undergo Limb Amputation than Those with PAD and Type 2 Diabetes
Source: J Clin Med. 2020 Aug 31;9(9):2809. doi: 10.3390/jcm9092809 (PMC7563979; doi:10.3390/jcm9092809)
Supplement: Supplementary file 1 [file jcm-09-02809-s001.pdf]

Supplemental Table S1: ICD-9 diagnosis codes for PAD diagnosis and other comorbidities	Corresponding ICD-9 code
Atherosclerosis of native arteries of extremities with intermittent claudication, rest pain, ulceration, gangrene, or unspecified symptoms	440.2 (440.20-440.24, 440.29)
Atherosclerosis of bypass graft of extremities	4409.3 (440.0-440.32)
Atherosclerosis-generalized or unspecified	440.9
Atherosclerosis of aorta	440.0
Diabetes mellitus with peripheral circulatory disorders	249.70, 249.71, 250.70-250.73
Peripheral angiopathy in other diseases	443.81
Peripheral vascular disease, unspecified	443.9
Buerger's disease	443.1
Arterial embolism/thrombosis of lower extremities of iliofemoral artery	444.22, 444.81
Intermittent claudication	440.21
Atrial fibrillation	427.31
History of myocardial infarction	412
History of percutaneous coronary intervention	V45.82
History of coronary artery bypass graft	V45. 81
History of cerebrovascular accident	430,431,434,436
Hypertension, congestive heart failure, chronic pulmonary disease, chronic kidney disease	Elixhauser comorbidities available within database
Hyperlipidemia	53
End Stage Renal Disease	3995, 5498, 5856
Smoking	V15.82, 305.1
Carotid artery disease	433.10
Ulcer	70710, 70714-15, 70719, 44023
Complicated Ulcer: defined as presence of ulcer with one of the following: gangrene, cellulitis, osteomyelitis	
Gangrene	785.4, 0400, 44024
Osteomyelitis	73007, 73017, 73027, 73097
Cellulitis	6807, 6827, 68100, 6819

Supplemental Table S2: ICD-9 diagnosis codes for Chronic Limb threatening Ischemia	Corresponding ICD-9 code
CLI if the following diagnosis code in the presence of PAD code:	
1. Gangrene	1. 785.4
2. Ulcer of lower limb	2. 707.10-707.15,707.19
3. Acute osteomyelitis of pelvic region and thigh	3. 730.05
4. Acute osteomyelitis of lower extremity	4. 730.06
5. Acute osteomyelitis of ankle and foot	5. 730.07
6. Chronic osteomyelitis of pelvic region and thigh	6. 730.15
7. Chronic osteomyelitis of lower extremity	7. 730.16
8. Chronic osteomyelitis of ankle and foot	8. 730.17
9. Cellulitis of lower extremity except foot	9. 682.6
10. Cellulitis of foot except toes	10. 682.7
11. Cellulitis of toes	11. 681.1

Supplemental Table S3: ICD-9 procedural codes	Corresponding ICD-9 code
Endovascular revascularization:	
1. Angioplasty or atherectomy of other noncoronary vessel(s)	1. 39.50
2. Insertion of noncoronary stent(s) or stent graft(s)	2. 39.90
Open revascularization:	
1. Aorta-iliac-femoral bypass	1. 39.25
2. Endarterectomy of lower limb artery	2. 38.18
3. Other (peripheral) vascular shunt or bypass	3. 39.29
1. Major Amputation	1. 84.14-15, 84.17
2. Minor Amputation	2. 84.10-84.12

Supplemental Table S4. The adjusted odds ratio and 95% confidence intervals of the covariates included in the multivariable unconditional logistic regression model for the outcome of Amputations

Covariate	Adjusted odds ratio	*p*-value	95% CI	
Type 1 Diabetes vs. Type 2 Diabetes	1.12	<.001	1.08	1.16
18-40	Ref			
41-60	1.04	0.127	0.99	1.09
61-75	0.93	0.003	0.88	0.97
>75	0.78	<.001	0.74	0.82
Female	0.75	<.001	0.73	0.76
Insurance type				
Public	Ref			
Private	1.12	<.001	1.10	1.15
Other	1.39	<.001	1.31	1.48
Race				
White	Ref			
African American	1.46	<.001	1.42	1.50
Other	1.17	<.001	1.13	1.21
Calendar year	0.97	<.001	0.97	0.98
Congestive heart failure	1.15	<.001	1.13	1.17
Chornic Lung disease	0.76	<.001	0.74	0.77
Hypertension	1.14	<.001	1.12	1.16
Chronic Kidney disease	0.98	0.11	0.97	1.00
Smoking	0.99	0.534	0.97	1.01
History of MI	0.99	0.599	0.97	1.02
History of PCI	0.78	<.001	0.75	0.80
History of CABG	0.91	<.001	0.89	0.93
Atrial fibrillation	0.88	<.001	0.86	0.90
Carotid artery disease	0.61	<.001	0.57	0.64
Dyslipidemia	0.82	<.001	0.80	0.83

History of CVA	0.99	0.48	0.97	1.02
Complicated Ulcer	2.34	<.001	2.29	2.39
CLTI	24.68	<.001	23.87	25.52
ESRD	1.12	<.001	1.09	1.15
IC	0.29	<.001	0.26	0.32

MI, myocardial infarction; PCI, percutaneous coronary intervention; CABG, coronary artery bypass graft; CVA, cerebrovascular accident; CLTI, chronic limb threatening ischemia; ESRD, end-stage renal disease; IC, intermittent claudication

Supplemental Table S5. The adjusted odds ratio and 95% confidence intervals of the covariates included in the multivariable unconditional logistic regression model for the outcome of Major Amputations

	**Odds Ratio**	***p*-value**	**95%CI**	
Type 1 Diabetes vs. Type 2 Diabetes	1.15	<.001	1.11	1.20
Age groups, years				
18-40	Ref			
41-60	1.28	<.001	1.20	1.37
61-75	1.26	<.001	1.18	1.35
>75	1.21	<.001	1.13	1.30
Female	0.85	<.001	0.84	0.87
Insurance type				
Public	Ref			
Private	0.89	<.001	0.86	0.91
Other	1.02	0.72	0.93	1.10
Race				
White	Ref			
African American	1.61	<.001	1.56	1.66
Other	1.17	<.001	1.13	1.22
Calendar year	0.95	<.001	0.95	0.96
Congestive heart failure	1.38	<.001	1.35	1.41
Chronic Lung disease	0.85	<.001	0.83	0.87
Hypertension	1.13	<.001	1.11	1.16
Chronic Kidney disease	1.06	<.001	1.04	1.08
Smoking	1.01	0.55	0.98	1.04
History of MI	1.00	0.83	0.96	1.03
History of PCI	0.75	<.001	0.72	0.78
History of CABG	0.93	<.001	0.90	0.95
Atrial fibrillation	0.96	<.001	0.94	0.99
Carotid artery disease	0.60	<.001	0.55	0.64
Dyslipidemia	0.75	<.001	0.73	0.76

History of CVA	1.22	<.001	1.18	1.25
Complicated Ulcer	1.33	<.001	1.30	1.36
CLTI	16.37	<.001	15.75	17.00
ESRD	1.30	<.001	1.26	1.34
IC	0.34	<.001	0.30	0.39

MI, myocardial infarction; PCI, percutaneous coronary intervention; CABG, coronary artery bypass graft; CVA, cerebrovascular accident; CLTI, chronic limb threatening ischemia; ESRD, end-stage renal disease; IC, intermittent claudication
